# Dysmyelination by Oligodendrocyte‐Specific Ablation of *Ninj2* Contributes to Depressive‐Like Behaviors

**DOI:** 10.1002/advs.202103065

**Published:** 2021-11-17

**Authors:** Yuxia Sun, Xiang Chen, Zhimin Ou, Yue Wang, Wenjing Chen, Tongjin Zhao, Changqin Liu, Ying Chen

**Affiliations:** ^1^ State Key Laboratory of Cellular Stress Biology School of Life Sciences Xiamen University Xiamen Fujian 361005 China; ^2^ Shanghai Key Laboratory of Metabolic Remodeling and Health Institute of Metabolism and Integrative Biology Zhongshan Hospital Fudan University Shanghai 200438 China; ^3^ Department of Endocrinology and Diabetes The First Affiliated Hospital of Xiamen University Fujian Province Key Laboratory of Diabetes Translational Medicine Xiamen Fujian 361101 China

**Keywords:** depression, necroptosis, Ninj2, oligodendrocytes

## Abstract

Depression is a mental disorder affecting more than 300 million people in the world. Abnormalities in white matter are associated with the development of depression. Here, the authors show that mice with oligodendrocyte‐specific deletion of *Nerve injury‐induced protein 2 （Ninj2)* exhibit depressive‐like behaviors. Loss of *Ninj2* in oligodendrocytes inhibits oligodendrocyte development and myelination, and impairs neuronal structure and activities. Ninj2 competitively inhibits TNF*α*/TNFR1 signaling pathway by directly binding to TNFR1 in oligodendrocytes. Loss of *Ninj2* activates TNF*α*‐induced necroptosis, and increases C‐C Motif Chemokine Ligand 2 (Ccl2) production, which might mediate the signal transduction from oligodendrocyte to neurons. Inhibition of necroptosis by Nec‐1s administration synchronously restores oligodendrocyte development, improves neuronal excitability, and alleviates depressive‐like behaviors. This study thus illustrates the role of Ninj2 in the development of depression and myelination, reveals the relationship between oligodendrocytes and neurons, and provides a potential therapeutic target for depression.

## Graphical abstract

Oligodendrocyte‐specific deletion of *Nerve injury‐induced protein 2 （* *Ninj2)* leads to depressive‐like behaviors in mice. Loss of *Ninj2* inhibits oligodendrocyte development and myelination by inducing TNF*α*‐mediated necroptosis, impairs neuron functions, and results in depression. Inhibition of necroptosis synchronously restores oligodendrocyte development and alleviates depressive‐like behaviors. The findings thus reveal the importance of oligodendrocytes in depression.

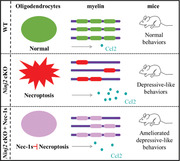

## 1 Introduction

Depression is a common mental disorder that affects more than 300 million people in the world. The World Health Organization has predicted that major depressive disorder will become the 1st ranking global burden of disease by 2030.^[^ ^1^ ^]^ The causation of depression is multifactorial.^[^ ^2^ ^]^ Abnormal neurogenesis is highly associated with the development of depression.^[^ ^3^ ^]^ In central nervous system (CNS), oligodendrocytes‐formed myelin sheath is the major component of the white matter, and it is essential for normal neuronal functions. The ensheathment of myelin around the axons allows rapid saltatory impulse propagation along the nerve fibers.^[^ ^4^ ^]^ Furthermore, oligodendrocytes metabolically support the neurons and facilitate neuron development.^[^ ^5^ ^]^ Oligodendrocyte development is a highly regulated process controlled by intrinsic factors including chromatin remodeling, transcriptional regulation, and post‐translational modifications,^[^ ^6^ ^]^ or extrinsic factors such as neurotransmitters and the metabolites released from the nearby neurons.^[^ ^7^ ^]^ Recently, impaired white matter integrity has been connected to the development of depression.^[^ ^8^ ^]^ However, further studies are in need to show the mechanism underpinning this connection.

Nerve injury‐induced protein 2 (Ninj2), a membrane adhesion molecule, was first found in the myelin‐forming Schwann cells in the peripheral nervous system. It induces neurite outgrowth by promoting homophilic cellular interaction.^[^ ^9^ ^]^ In CNS, *Ninj2* is predominantly expressed in the oligodendrocyte lineage cells,^[^ ^10^ ^]^ however, its function remains elusive. Single nucleotide polymorphisms studies points out that mutations of *Ninj2* could be risk factors of ischemic stroke and multiple sclerosis,^[^ ^11^ ^]^ and even associated with suicide attempts.^[^ ^12^ ^]^ Recent studies indicate that the expression of *Ninj2* is downregulated in mice with stress‐induced depression.^[^ ^13^ ^]^ However, whether and how Ninj2 plays a role in the development of depression is largely unknown.

In our present study, to demonstrate the function of Ninj2 in depression, we generated oligodendrocyte‐specific *Ninj2*‐knockout mice to evaluate the effect of Ninj2 in depression. We found that oligodendrocyte dysfunction was closely associated with depression. Loss of *Ninj2* in oligodendrocytes stimulated TNF*α*‐induced necroptosis, hence simultaneously inhibited myelination and led to depressive‐like behaviors. Mechanistically, the production of C‐C Motif Chemokine Ligand 2 (Ccl2) was significantly induced during the activation of necroptosis in the *Ninj2*‐deficient oligodendrocytes, and it might mediate the conversation between oligodendrocytes and neurons. These mechanistic insights into the modulation of depression by Ninj2 broadened the biological significance of oligodendrocytes in CNS, and provided a therapeutic target for depression in clinical studies.

## 2 Results

### 2.1 Oligodendrocyte‐Specific Knockout of *Ninj2* Leads to Depressive‐Like Behaviors

Accumulating evidences have indicated that myelin is associated with depression. Regenold et al. showed that unipolar major depression subjects have reduced white matter intensity compared with normal control subjects.^[^ ^14^ ^]^ In chronic social defeat mice showing depressive‐like behaviors, the expression levels of the myelin‐related genes are largely downregulated.^[^ ^15^ ^]^ Furthermore, a single‐nucleus transcriptomic analysis of the prefrontal cortex in major depressive disorder implicate that oligodendrocyte precursor cells (OPCs) plays a role in depression.^[^ ^16^ ^]^ To investigate the role of oligodendrocytes during the development of depression, we generated an *Olig1^cre/+^;Gi‐DREADD* mice strain, in which the activity of oligodendrocytes was inhibited upon clozapine‐*N*‐oxide (CNO) treatment (Figure S1A, Supporting Information). Using this strain, we performed behavioral tests to examine whether inhibition of oligodendrocyte activity would lead to depressive‐like behaviors. As shown, when the *Olig1^cre/+^;Gi‐DREADD* mice were treated with CNO, they exhibited longer immobility durations in tail suspension test (TST) and forced swimming test (FST) than control mice (Figure S1B,C, Supporting Information). Meanwhile, the CNO‐treated *Olig1^cre/+^;Gi‐DREADD* mice showed reduced sucrose preference (Figure S1D, Supporting Information). The depressive‐like behaviors in the CNO‐treated *Olig1^cre/+^;Gi‐DREADD* mice clearly reflected that dysfunction in oligodendrocytes was sufficient to trigger depression.

Next, we were prompted to investigate the key genes, which were predominantly expressed in oligodendrocytes, and potentially involved in the development of depression. We analyzed two published datasets that contained the transcriptomic data of the mice showing depressive behaviors. In a chronic social defeat stress‐induced depression mice model, RNA‐seq analysis in their cortex samples indicated that the expressions of 5083 genes were significantly changed (fold change > 1.5, or < 0.67).^[^ ^13b^ ^]^ In the other dataset obtained from the cortex samples of the mice with chronic variable stress‐induced depression, the expression levels of 3857 genes were greatly changed,^[^ ^13a^ ^]^ compared to control mice. After alignment, 171 common genes were found in both datasets. According to the gene expression pattern of the cortex,^[^ ^10^ ^]^ 25 of those common genes were substantially expressed in the cell lineages in CNS, and *Ninj2* was the only gene that is predominantly expressed in oligodendrocyte lineages. Therefore, we chose *Ninj2* as the candidate, to explore its role in the development of depression.

**Figure 1:**
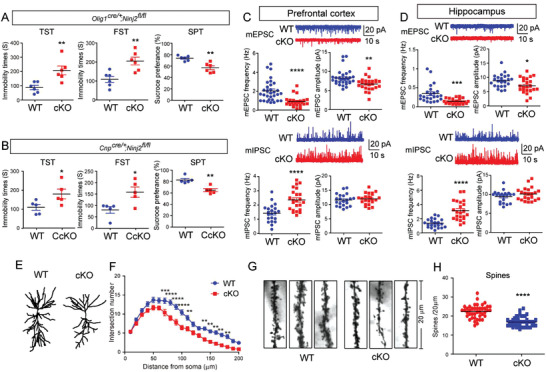
Loss of *Ninj2* in oligodendrocytes leads to depressive‐like behaviors. A,B) Tail suspension test (TST), force swimming test (FST), and sucrose preference test (SPT), were performed in *Olig1^cre/+^;Ninj2^fl/fl^* mice (cKO) (*n* = 6 mice/genotype) (A) or *Cnp^cre/+^;Ninj2^fl/fl^* mice (CcKO) (*n* = 5 mice/genotype) (B) and their littermate controls. C,D) Whole‐cell patch‐clamp recording was performed to show the representative traces and quantification of frequencies and amplitudes of mEPSCs or mIPSCs from prefrontal cortex pyramidal neurons (mEPSCs: *n* = 31 neurons for WT mice, *n* = 25 neurons for cKO mice; mIPSCs: *n* = 21 neurons for WT mice, *n* = 23 neurons for cKO mice; from six mice/genotype) (C), or hippocampal CA1 pyramidal neurons (mEPSCs: *n* = 23 neurons for WT mice, *n* = 24 neurons for cKO mice; mIPSCs: *n* = 21 neurons for WT mice, *n* = 22 neurons for cKO mice; from six mice/genotype) (D). E–H) WT and cKO mice were subjected to Golgi staining. Sholl analysis of dendritic complexity in hippocampal CA1 pyramidal neurons (*n* = 28 neurons from three mice for each group) was shown in (E,F), Apical distal spine numbers of the pyramidal neurons in hippocampal CA1 regions (*n* = 42 dendritic segments for WT mice, *n* = 49 dendritic segments for cKO mice, from three mice/genotype) were shown in (G,H), Scale bars = 20 µm. For all panels, mice were subjected to experiments at P60. All the quantification data are presented as mean ± SEM, *p*‐values are calculated using two‐tailed unpaired Student's *t*‐test (A–D,H), or two‐way ANOVA with Tukey's multiple comparisons test (F), F (interaction [*F* (19, 1080) = 2.014, *p* = 0.0061]),*p < 0.05, **p < 0.01, ***p < 0.001, ****p < 0.0001.

We then generated two oligodendrocyte‐specific *Ninj2* deletion mice strains (*Olig1^cre/+^;Ninj2^fl/fl^* and *Cnp^cre/+^;Ninj2^fl/fl^*) by crossing the *Olig1^cre/+^* or *Cnp^cre/+^* with *Ninj2^fl/fl^* mice strains (Figure S2, Supporting Information). Through a series of behavioral tests, we found that *Ninj2*‐deficient mice showed longer immobility durations in TST and FST, and reduced sucrose preference, compared with their WT counterparts (**Figure** **1**A,B), suggesting that loss of *Ninj2* in oligodendrocytes resulted in depressive‐like behaviors, similar to what we observed in the CNO‐treated *Olig1^cre/+^;Gi‐DREADD* mice. At the same time, the *Ninj2*‐deficient mice did not show any difference in Morris Water Maze test, Y maze test, Novel object recognition test, elevated plus maze test, or the open field test, compared with the WT mice (Figure S3, Supporting Information). These results suggested that loss of *Ninj2* in oligodendrocytes had no significant effect on memory, recognition, or anxiety‐like behaviors.

To show the physiological impact of *Ninj2* deletion, we detected the pyramidal neurons functions in the prefrontal cortex and hippocampus CA1 area by whole‐cell patch‐clamp recording. As shown, *Olig1^cre/+^;Ninj2^fl/fl^* mice had reduced frequencies and amplitudes of miniature excitatory postsynaptic currents (mEPSCs), but augmented frequencies of miniature inhibitory postsynaptic currents (mIPSCs) in both regions (Figure 1C,D), suggesting the neuronal dysfunction in *Ninj2*‐deficient mice. Dendritic complexity was closely related with neuronal function, and impaired synaptic plasticity was also associated with depression.^[^ ^17^ ^]^ Furthermore, using Golgi staining, we found that the pyramidal neurons in the hippocampus CA1 area of *Olig1^cre/+^;Ninj2^fl/fl^* mice were less developed, as they showed significantly reduced dendritic complexity, as well as reduced density of dendritic spines, when compared with their WT counterparts (Figure 1E–H and Figure S4, Supporting Information). These results supported our hypothesis that *Ninj2* deficiency in oligodendrocytes changed neuronal structure and further caused neuronal dysfunctions, resulting in depressive‐like behaviors.

### 2.2 Loss of *Ninj2* Inhibits Myelinogenesis

**Figure 2:**
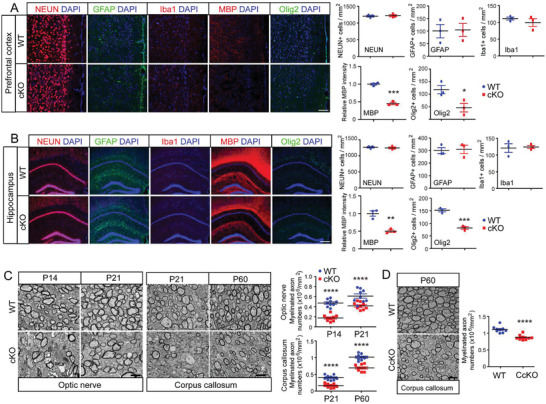
Loss of *Ninj2* in oligodendrocytes inhibits myelination. A,B) Immunofluorescent staining against NEUN, GFAP, Iba1, MBP, and Olig2 in the prefrontal cortex (A) or hippocampal CA1 (B) sections from WT or *Olig1^cre/+^;Ninj2^fl/fl^* (cKO) mice. The quantification of NEUN^+^ GFAP^+^, Iba1^+^, and Olig2^+^ cell numbers, and the relative MBP intensity, were shown in the right panel. Scale bar = 100 µm (A), 250 µm (B). *n* = 3 mice/genotype. C) Electron microscopic examination of the optic nerve at P14 and P21 and corpus callosum at P21 and P60 from WT or cKO mice. Scale bar = 2 µm. *n* = 3 mice/genotype. D) Electron microscopic examination of the corpus callosum at P60 from WT or *Cnp^cre/+^;Ninj2^fl/fl^* (CcKO) mice. The quantified myelinated axon numbers were shown on the right panel. Scale bar = 2 µm. *n* = 3 mice/genotype. All the quantification data are presented as mean ± SEM, *p*‐values are calculated using two‐tailed unpaired Student's *t*‐test, *p < 0.05, **p < 0.01, ***p < 0.001, ****p < 0.0001.

Recently, the linkages between oligodendrocytes and mental disorders have been reported.^[^ ^18^ ^]^ Since *Ninj2* was predominantly expressed in oligodendrocytes, we hypothesized that loss of *Ninj2* caused depressive‐like behaviors through its modulation in oligodendrocytes. To test our hypothesis, we examined the oligodendrocyte development and myelination status in the *Olig1^cre/+^;Ninj2^fl/fl^* mice. Through immunofluorescent imaging, we found that loss of *Ninj2* reduced MBP^+^ area and Olig2^+^ oligodendrocytes in both prefrontal cortex and hippocampus CA1 area, but had no effect on the population of NeuN^+^ neurons, GFAP^+^ astrocytes, or Iba1^+^ microglia (**Figure** **2**A,B), suggesting that loss of *Ninj2* in oligodendrocytes primarily inhibited oligodendrocyte development.

Consistent with the suppressed oligodendrocyte development, we observed dysmyelination phenotypes in the *Ninj2* deficient mice. Ultrastructural analyses on optic nerve and corpus callosum showed that *Olig1^cre/+^;Ninj2^fl/fl^* mice had significantly lower myelinated axon numbers, compared with WT mice (Figure 2C). Moreover, the MBP intensity and the protein levels of MBP and CNP in the corpus callosum were significantly reduced by *Ninj2* ablation throughout the duration of myelin development (Figure S5, Supporting Information). Consistently, in *Cnp^cre/+^;Ninj2^fl/fl^* mice, the myelinated axon number in the corpus callosum was also lower than that in WT mice (Figure 2D). However, loss of *Ninj2* had no effect on G‐ratio (Figure S6, Supporting Information). The hypomyelination phenotype in these *Ninj2*‐deficient mice was further corroborated by their motor defects. Both strains showed significant reductions in their grip strength and motor coordination (Figure S7, Supporting Information).

### 2.3 *Ninj2* Ablation in Oligodendrocytes Promotes Canonical Necroptosis

**Figure 3:**
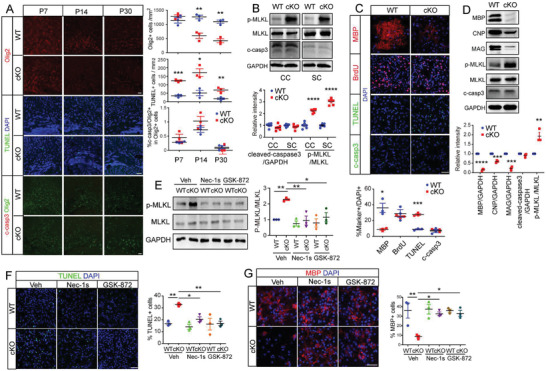
Ablation of *Ninj2* in oligodendrocytes induces necroptosis. A) Immunofluorescent staining against Olig2, TUNEL, and cleaved‐caspase3 were performed in the corpus callosum sections from WT or *Olig1^cre/+^;Ninj2^fl/fl^* (cKO) mice at P7, P14, and P30, the number of the Olig2^+^, TUNEL^+^ cells, or the percentage of cleaved‐caspase3^+^ cells in Olig2^+^ cells, were quantified and shown on the right panel. Scale bar = 50 µm. *n* = 3 mice/genotype. B) Western blotting analyses against p‐MLKL, MLKL, and cleaved‐caspase3 on the corpus callosum or spinal cord samples from WT or cKO mice at P7. Densitometric quantification of p‐MLKL/MLKL or cleaved‐caspase3/GAPDH ratio was shown at the bottom panel. *n* = 6 mice/genotype. C) Immunofluorescent staining against MBP, BrdU, TUNEL, and cleaved‐caspase3 in cultured oligodendrocytes from WT or cKO mice, the percentages of MBP^+^, BrdU^+^, TUNEL^+^, and cleaved‐caspase3^+^ cells were shown at the bottom panel. Scale bar = 50 µm. *n* = 3 independent experiments. D) Western blot assays with the indicated antibodies in oligodendrocytes from WT or cKO mice. Densitometric quantification of p‐MLKL/MLKL or MBP, CNP, MAG, cleaved‐caspase3/GAPDH ratio was shown at the bottom panel. *n* = 3 independent experiments. E–G) Oligodendrocytes from WT or cKO mice were treated with vehicle, Nec‐1s (1 µm) or GSK‐872 (0.25 µM) for 48 h respectively, and then subjected to western blot analyses against the indicated proteins (E) or immunofluorescent staining against TUNEL (F) and MBP (G). Densitometric quantification of p‐MLKL/MLKL ratio was shown at the right panel of (E). The percentages of the TUNEL^+^ or MBP^+^ in total DAPI^+^ cells were quantified and shown on the right panels of (F) and (G). Scale bar = 50 µm. *n* = 3 independent experiments for each group. All the quantification data are presented as mean ± SEM, *p*‐values are calculated using two‐tailed unpaired Student's *t*‐test (A–D), or two‐way ANOVA with Tukey's multiple comparisons test (E–G), E (Nec‐1s: interaction [*F*_1,8_ = 10.35, *p* = 0.0123]; GSK‐872: interaction [*F*_1,8_ = 5.37, *p* = 0.0491]), F (Nec‐1s: interaction [*F*_1,8_ = 8.443, *p* = 0.0197], GSK‐872: interaction [*F*_1,8_ = 7.957, *p* = 0.0225]), G (Nec‐1s: interaction [*F*_1,8_ = 5.520, *p* = 0.0467], GSK‐872: interaction [*F*_1,8_ = 7.913, *p* = 0.0227]), *p < 0.05, **p < 0.01, ***p < 0.001, ****p < 0.0001.

To explore molecular mechanisms underpinning with the dysmyelination observed upon oligodendrocytic *Ninj2* deletion, we used immunofluorescent imaging to evaluate the effect of Ninj2 on oligodendrocyte development, and found that ablation of *Ninj2* substantially reduced the total number of Olig2^+^ oligodendrocytes at P14 and P30 in corpus callosum (**Figure** **3**A). At the same time, upon *Ninj2* deletion, an increased population of TUNEL^+^ cells was observed, but the proportion of CC1^+^, PDGFR*α* ^+^, Ki67^+^ and cleaved‐caspase3^+^ cells in the total Olig2^+^ oligodendrocytes at the indicated time points were unaffected (Figure 3A and Figure S8, Supporting Information), suggesting that loss of *Ninj2* primarily induced nonapoptotic cell death in oligodendrocytes. Furthermore, western blot analysis of corpus callosum and spinal cord samples showed that loss of *Ninj2* increased p‐MLKL level, a surrogate marker for cell necroptosis, but had no effect on cleaved‐caspase3 level (Figure 3B). All these data suggested that loss of *Ninj2* in oligodendrocytes reduced the population of Olig2^+^ oligodendrocytes by inducing necroptotic cell death.

We then went on to confirm the results in cultured mouse oligodendrocytes. Consistently, ablation of *Ninj2* greatly reduced the number of MBP^+^ oligodendrocytes, and inhibited MBP, CNP, and MAG level (Figure 3C,D). However, loss of *Ninj2* had no effect on oligodendrocyte proliferation (Figure 3C). Furthermore, loss of *Ninj2* induced necroptotic cell death, but had no effect on apoptosis (Figure 3C,D), which further confirmed that *Ninj2* ablation induced necroptosis in oligodendrocytes.

It has been previously reported that canonical necroptosis, mediated by RIPK1 and RIPK3, played an important role in cell death in oligodendrocytes.^[^ ^19^ ^]^ We then wondered that whether blocking the canonical necroptosis pathway could rescue oligodendrocyte development. We treated primary cultured oligodendrocytes with Nec‐1s and GSK‐872, the inhibitors targeting RIPK1 and RIPK3, respectively.^[^ ^20^ ^]^ We found that Nec‐1s or GSK‐872 treatment decreased intracellular p‐MLKL level in the *Ninj2‐*knockout oligodendrocytes (Figure 3E). Moreover, it also reduced the population of TUNEL^+^ cells (Figure 3F), and increased the population of MBP^+^ cells in the *Ninj2*‐deficient oligodendrocytes (Figure 3G). Collectively, our data indicate that loss of *Ninj2* inhibits oligodendrocyte development and myelination due to its inductive effect on necroptosis.

### 2.4 Ninj2 Interrupts TNF*α*/TNFR1 Signaling

**Figure 4:**
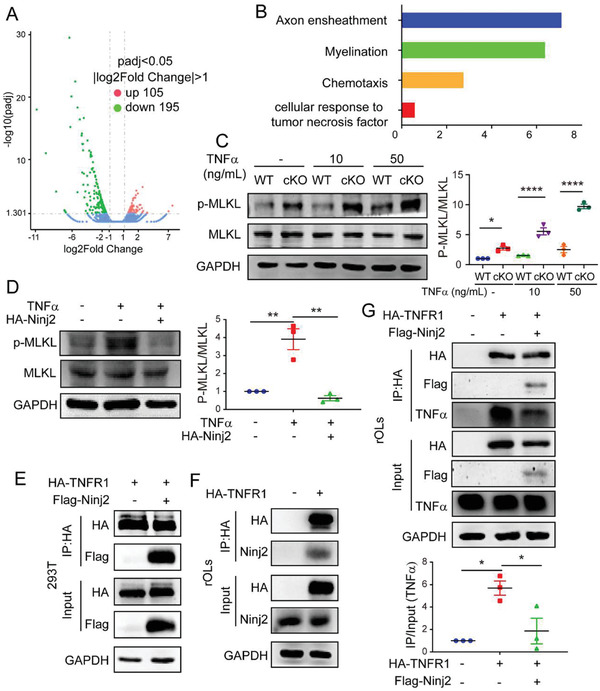
Ninj2 inhibits TNF*α*‐induced necroptosis through interaction with TNFR1. A) Volcano diagram showing the genes whose expression level was significantly changed in the oligodendrocytes from *Olig1^cre/+^;Ninj2^fl/fl^* (cKO) mice. B) Gene Ontology (GO) analysis showing the biological processes in which the significantly changed genes involved. C,D) Western blotting analyses against p‐MLKL, MLKL in the oligodendrocytes from WT or cKO mice that treated with vehicle, 10 or 50 ng mL^−1^ of TNF*α* for 48 h, respectively (C), or in oligodendrocytes transfected with empty or *Ninj2*‐overexpressing vectors, and then treated with 100 ng mL^−1^ of TNF*α* for 48 h (D). Densitometric quantification of p‐MLKL/MLKL ratio from at least three independent assays is shown at the right panel of (C) and (D). E) Co‐immunoprecipitation analysis in HEK293T cells that transfected with *Ninj2* and *TNFR1*‐overexpressing vectors, alone or in combination as indicated. *n* = 3 independent experiments. F) Co‐immunoprecipitation analysis in rat oligodendrocytes that transfected with empty or *TNFR1*‐overexpressing vectors. *n* = 3 independent experiments. G) Co‐immunoprecipitation analysis in rat oligodendrocytes that transfected with empty, *Ninj2* or *TNFR1*‐overexpressing vectors, alone or in combination as indicated. Densitometric quantification of IP/Input (TNF*α*) ratio from at least three independent assays was shown at the bottom panel. All the quantification data are presented as mean ± SEM, *p*‐values are calculated using two‐way ANOVA (C) or one‐way ANOVA with Tukey's multiple comparisons test (D,G), C (interaction [*F*_2,12_ = 26.56, *p* < 0.0001]), D (interaction [*F*_2,6_ = 27.31, *p* = 0.001]), G (interaction [*F*_2,6_ = 10.91, *p* = 0.01]), *p < 0.05, **p < 0.01, ****p < 0.0001.

In order to elucidate the molecular basis underlying the necroptosis induced by *Ninj2* deletion, we performed RNA‐Seq analysis of WT or *Ninj2*‐deficient mouse oligodendrocytes. The analysis showed that *Ninj2* deletion in oligodendrocytes induced the expression of 105 genes (fold change > 2), and reduced the expression of 195 genes (to at least 50%) (**Figure** **4**A). According to Gene Ontology analysis, the downregulated genes were majorly involved in axon ensheathment or myelination, and the upregulated genes were found in chemotaxis and cellular response to tumor necrosis factor (Figure 4B). These results hinted that Ninj2 might play a role in TNF*α* signaling, a well‐known pathway to trigger necroptosis.^[^ ^21^ ^]^

To study the potential role of Ninj2 in TNF*α* signaling, we first examined the cellular response to TNF*α* in WT or *Ninj2*‐deficient oligodendrocytes. As shown in Figure 4C, in the presence of TNF*α* treatment, the level of p‐MLKL was induced at a dose‐dependent manner in both WT and *Olig1^cre/+^;Ninj2^fl/fl^* oligodendrocytes, but the level of p‐MLKL increased more dramatically in the TNF*α*‐treated *Ninj2*‐deficient cells, compared to the WT cells. Similarly, in cultured rat oligodendrocytes, TNF*α* sufficiently induced intracellular p‐MLKL, but it was greatly abolished by *Ninj2* overexpression (Figure 4D). We then speculated that Ninj2 could interrupt TNF*α*/TNFR1 binding. To test the speculation, co‐immunoprecipitation assays in HEK293T cells were performed and showed that Ninj2 directly interacted with TNFR1 (Figure 4E). In rat oligodendrocytes, similar interaction between Ninj2 and TNFR1 was observed (Figure 4F). In addition, Ninj2/TNFR1 interaction interrupted the recruitment of TNF*α* to TNFR1. Overexpression of *Ninj2* in oligodendrocytes attenuated the interaction between TNFR1 and the endogenous TNF*α* (Figure 4G). Collectively, our data illustrate that Ninj2 competitively inhibits TNF*α*/TNFR1 signaling through interaction with TNFR1.

### 2.5 *Ninj2* Ablation Increases Ccl2 Production in Oligodendrocytes

**Figure 5:**
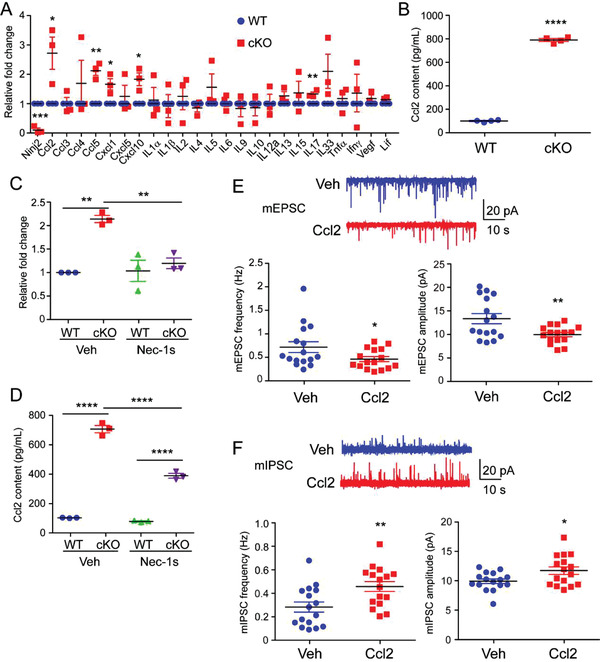
Ccl2 release from necroptotic oligodendrocytes changes the activities of primary pyramidal neurons. A) Expression of inflammatory cytokines in oligodendrocytes derived from WT or *Olig1^cre/+^;Ninj2^fl/fl^* (cKO) mice. *n* = 3 independent experiments. B) Ccl2 levels in oligodendrocytes cell culture supernatant derived from WT or cKO mice was determined by ELISA. *n* = 4 independent experiments. C) Ccl2 mRNA levels in primary oligodendrocytes from WT or cKO mice treated with vehicle or Nec‐1s (1 µm). *n* = 3 independent experiments. D) Ccl2 levels in oligodendrocytes cell culture supernatant derived from WT or *Olig1^cre/+^;Ninj2^fl/fl^* (cKO) mice treated with vehicle or Nec‐1s (1 µm) was determined by ELISA. *n* = 3 independent experiments. E,F) Whole‐cell patch‐clamp recording was performed to show the representative traces and quantification of frequencies and amplitudes of mEPSCs or mIPSCs from primary culture pyramidal neurons treated with vehicle or Ccl2 (100 ng mL^−1^) for 48 h. (*n* = 16 neurons for each group). All the quantification data are presented as mean ± SEM, *p*‐values are calculated using two‐tailed unpaired Student's *t*‐test (A,B,E,F) or two‐way ANOVA with Tukey's multiple comparisons test (C,D), C (interaction [*F*_1,8_ = 13.76, *p* = 0.006]), D (interaction [*F*_1,8_ = 97.49, *p* < 0.0001]), *p < 0.05, **p < 0.01, ***p < 0.001, ****p < 0.0001.

As shown in Figure 4B, *Ninj2*‐deficient oligodendrocytes showed increased expression levels of genes involved in chemotaxis and cellular response to TNF*α*. As cytokines were tightly associated with the onset of depression,^[^ ^22^ ^]^ we tested the expression levels of inflammatory cytokines and found that *Ccl2* was the most significantly upregulated in the *Ninj2*‐deficient oligodendrocytes, compared to the WT cells (**Figure** **5**A). Furthermore, Ccl2 content increased in conditioned media from *Ninj2*‐deficient oligodendrocytes (Figure 5B). Ccl2 production was increased during the process of necroptosis.^[^ ^23^ ^]^ When we used Nec‐1s to inhibit oligodendrocytic necroptosis, the mRNA level and content of Ccl2 in the conditioned media of *Ninj2*‐deficient oligodendrocytes greatly reduced (Figure 5C,D), providing additional evidence that the excessive Ccl2 production came from necroptosis triggered by TNF*α* signaling overactivation in *Ninj2‐*deficient oligodendrocytes. Previously, Ccl2 has been reported to affect the activities of different kinds of neurons.^[^ ^24^ ^]^ Blockage of Ccl2/CCR2 signaling attenuated neuropathic pain and depression.^[^ ^24c^ ^]^ Here, when the primary cultured pyramidal neurons were treated with Ccl2, whole‐cell patch‐clamp recording displayed that Ccl2 administration efficiently repressed the frequencies and amplitudes of mEPSCs, and induced the frequencies and amplitudes of mIPSCs (Figure 5E,F). These data provide a clue that the *Ninj2*‐deficient oligodendrocytes impair neuronal activities by its excessive Ccl2 secretion.

### 2.6 Inhibition of Cell Necroptosis in *Olig1^cre/+^;Ninj2^fl/fl^* Mice Rescues Myelination and Alleviated Depressive‐Like Behavior

**Figure 6:**
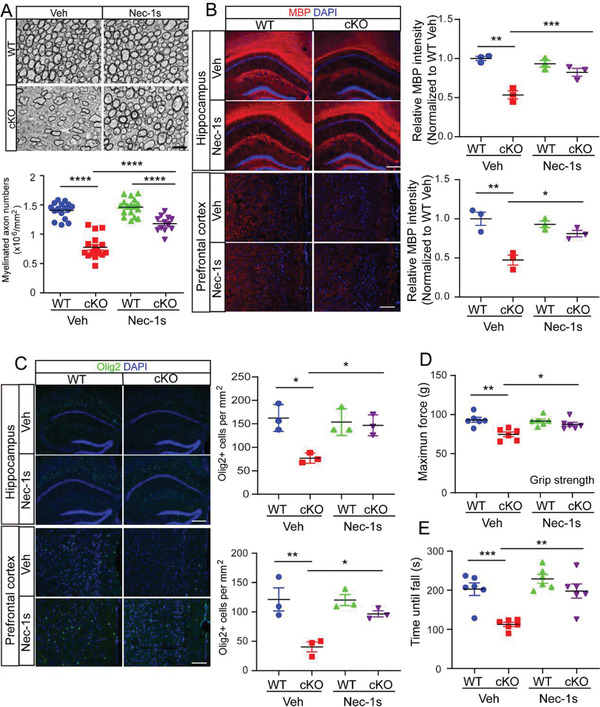
Nec‐1s treatment restores oligodendrocyte development and myelination. WT or *Olig1^cre/+^;Ninj2^fl/fl^* (cKO) mice received i.p. injection with vehicle or Nec‐1s (10 mg kg^−1^) from P60 to P90, and then subjected to the following experiments. A) Electron microscopic examination was performed in the corpus callosum, the myelinated axon numbers were quantified and shown on the bottom panel. Scale bar = 2 µm. *n* = 3 mice/group. B,C) Immunofluorescent staining against MBP and Olig2 was performed in the hippocampus CA1 and prefrontal cortex sections from WT and cKO mice. The quantification of the relative MBP intensity or Olig2^+^ cells was shown in the right panel. Scale bar = hippocampus CA1, 250 µm, prefrontal cortex, 100 µm. *n* = 3 mice/group. D,E) Forelimb grip strength test (D) and rotarod test (E) were performed, *n* = 6 mice/group. All the quantification data are presented as mean ± SEM, *p*‐values are calculated using two‐way ANOVA with Tukey's multiple comparisons test, A (interaction [*F*_1,58_ = 18.81, *p* < 0.0001]), B (MBP CA1: interaction [*F*_1,8_ = 16.50, *p* = 0.0036]; MBP PFC: interaction [*F*_1,8_ = 11.45, *p* = 0.0096]), C (Olig2 CA1: interaction [*F*_1,8_ = 8.327, *p* = 0.0203], Olig2 PFC: interaction [*F*_1,8_ = 5.692, *p* = 0.0441]), D (interaction [*F*_1,20_ = 6.294, *p* = 0.0208), E (interaction [*F*_1,20_ = 4.685, *p* = 0.0427]), *p < 0.05, **p < 0.01, ***p < 0.001, ****p < 0.0001.

To explore the physiological significance of protecting oligodendrocytes from necroptosis, we treated the mice with Nec‐1s to show its effects in vivo. Consistent with the findings in vitro, after Nec‐1s treatment, myelination in *Olig1^cre/+^;Ninj2^fl/fl^* mice was largely restored. In corpus callosum, their myelinated axon numbers significantly increased (**Figure** **6**A). In both hippocampus CA1 area and prefrontal cortex, the MBP^+^ area and Olig2^+^ numbers were also rescued, compared with the untreated mice (Figure 6B,C). In keeping with the improved myelination, the motor defects in *Olig1^cre/+^;Ninj2^fl/fl^* mice were also alleviated, as they had improved grip strength and motor coordination (Figure 6D,E).

**Figure 7:**
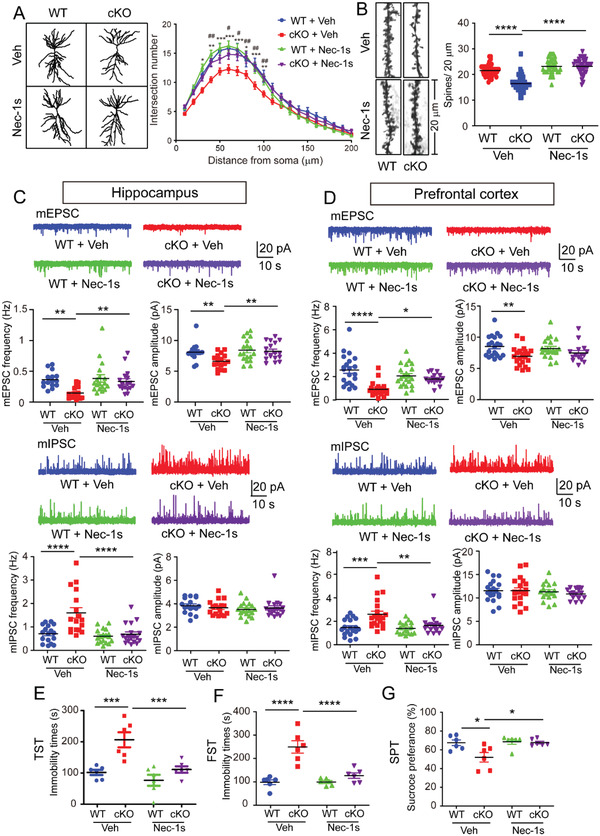
Nec‐1s treatment alleviates depressive‐like behaviors. WT or *Olig1^cre/+^;Ninj2^fl/fl^* (cKO) mice received i.p. injection with vehicle or Nec‐1s (10 mg kg^−1^) from P60 to P90, and then subjected to the following experiments. A,B) Mice were subjected to Golgi staining. Sholl analysis of dendritic complexity in hippocampal CA1 pyramidal neurons (*n* = 25 neurons for WT+Veh, *n* = 29 neurons for cKO+Veh, *n* = 32 neurons for WT+Nec‐1s, *n* = 30 neurons for cKO+Nec‐1s, from three mice/group) was shown in (A) (* indicated the significance of comparison between WT+Veh and cKO+Veh, # indicated the significance of comparison between cKO+Veh and cKO+Nec‐1s). Apical distal spine numbers of the pyramidal neurons in hippocampal CA1 regions (*n* = 41 dendritic segments for WT+Veh, *n* = 45 dendritic segments for cKO+Veh, *n* = 40 dendritic segments for WT+Nec‐1s, *n* = 41 dendritic segments for cKO+Nec‐1s, from 3 mice/group) were shown in (B). C,D) Whole‐cell patch‐clamp recording was performed to show the representative traces and quantification of frequencies and amplitudes of mEPSCs or mIPSCs from hippocampal CA1 pyramidal neurons (mEPSC: *n* = 15 neurons for WT+Veh, *n* = 17 neurons for cKO+Veh, *n* = 18 neurons for WT+Nec‐1s, *n* = 17 neurons for cKO+Nec‐1s; mIPSC: *n* = 17 neurons for WT+Veh, *n* = 17 neurons for cKO+Veh, *n* = 18 neurons for WT+Nec‐1s, *n* = 18 neurons for cKO+Nec‐1s; from 5 mice/group) (C), or prefrontal cortex pyramidal neurons (mEPSC: *n* = 21 neurons for WT+Veh, *n* = 21 neurons for cKO+Veh, *n* = 21 neurons for WT+Nec‐1s, *n* = 15 neurons for cKO+Nec‐1s; mIPSC: *n* = 19 neurons for WT+Veh, *n* = 20 neurons for cKO+Veh, *n* = 17 neurons for WT+Nec‐1s, *n* = 16 neurons for cKO+Nec‐1s; from 5 mice/group) (D). E–G) Tail suspension test (TST), forced swimming test (FST), and sucrose preference test (SPT), were performed to evaluate their depressive‐like behaviors (*n* = 6 mice/group). All the quantification data are presented as mean ± SEM, *p*‐values are calculated using two‐way ANOVA with Tukey's multiple comparisons test, A (interaction [*F*_57,2238_ = 1.683, *p* = 0.0012]), B (interaction [*F*_1,163_ = 35.64, *p* < 0.0001]), C (mEPSC frequency: interaction [*F*_1,63_ = 4.438, *p* = 0.0391]; mEPSC amplitude: interaction [*F*_1,63_ = 4.777, *p* = 0.0326]; mIPSC frequency: interaction [*F*_1,66_ = 9.326, *p* = 0.0033]; mIPSC amplitude: interaction [*F*_1,66_ = 0.9254, *p* = 0.3396]), D (mEPSC frequency: interaction [*F*_1,74_ = 11.25, *p* = 0.0013]; mEPSC amplitude: interaction [*F*_1,74_ = 1.488, *p* = 0.2264]; mIPSC frequency: interaction [*F*_1,68_ = 4.863, *p* = 0.0308]; mIPSC amplitude: interaction [*F*_1,68_ = 0.2138, *p* = 0.6453]). E (interaction [*F*_1,20_ = 7.317, *p* = 0.0136]), F (interaction [*F*_1,20_ = 16.71, *p* = 0.0006]), G (interaction [*F*_1,20_ = 4.841, *p* = 0.0397]), *p < 0.05, **p < 0.01, ***p < 0.001, ****p < 0.0001.

Accompanied with the improved myelination, the neuronal structure and activities were recovered in *Olig1^cre/+^;Ninj2^fl/fl^* mice after Nec‐1s treatment. In hippocampus CA1 area, the pyramidal neurons structure was restored, as indicated by the increased dendritic complexity, and density of spines (**Figure** **7**A,B and Figure S9, Supporting Information). Most importantly, the neuronal activities were recovered in both hippocampus CA1 area and prefrontal cortex, after Nec‐1s treatment. As shown, in both areas, the frequencies of mEPSCs were elevated, whereas the frequencies of mIPSCs were reduced, to similar ranges as that in WT mice (Figure 7C,D). As a result of the recoveries on neuronal activities, the depressive‐like behaviors in *Olig1^cre/+^;Ninj2^fl/fl^* mice significantly diminished. After Nec‐1s treatment, *Olig1^cre/+^;Ninj2^fl/fl^* mice had significantly reduced immobility times in TST and FST, and increased sucrose preference, compared with the vehicle‐treated counterparts (Figure 7E–G). Combining all these data, we show that inhibition on the oligodendrocytic necroptosis in *Ninj2*‐deficient mice is efficient to diminish their depressive‐like behaviors.

## 3 Discussion

In our current study, we found that *Ninj2* deletion in oligodendrocytes exhibited depressive‐like behaviors. Loss of *Ninj2* inhibited oligodendrocyte development by activating TNF*α*‐mediated cell necroptosis. The induced oligodendrocytic necroptosis impaired myelination, and simultaneously changed the neuronal structure and activities in the implicated depression‐related brain regions, the prefrontal cortex and hippocampus CA1 area. Conversely, inhibition of necroptosis effectively counteracted the phenotypes in these mice. Our findings established a substantial connection between oligodendrocytes and depression.

After data mining with the online database in the depression mice models,^[^ ^10^, ^13^ ^]^ we chose *Ninj2* as our research candidate, because of its predominant expression patterns in oligodendrocyte lineage cells. Until now, the function of Ninj2 in oligodendrocytes was still poorly studied. In our model, we found that Ninj2 protected oligodendrocytes from TNF*α*‐induced canonical necroptosis. Among the membrane‐located cytokine receptors activating canonical necroptosis, TNFR1 was the most abundantly expressed in oligodendrocytes.^[^ ^10^ ^]^ And our RNA‐Seq analysis showed that a large set of genes that are involved in TNF*α* signaling pathway was induced. Furthermore, a robust interaction between Ninj2 and TNFR1, and an inhibitory effect of Ninj2 on the recruitment of TNF*α* to TNFR1 were observed. Combining those evidences, we summarize that Ninj2 functions as a protector for oligodendrocytes from canonical necroptosis. Notably, as previously reported, oligodendrocytes were susceptible to necroptotic cell death.^[^ ^19^, ^25^ ^]^ According to the database reported by Zhang et al., the expression levels of TNF*α* and TNFR1 were both higher in oligodendrocytes than in neurons or astrocytes, which might partially contribute to the cell susceptibility to programmed cell death.^[^ ^10^ ^]^ In this study, our findings implied that the oligodendrocyte‐specific expressing Ninj2 could be a protective machinery for the cells during the course of development.

The importance of oligodendrocytes in mental health has been emerging. It has been reported that oligodendrocytes are involved in the pathologies of several mental illnesses, including depression,^[^ ^18a,b^ ^]^ bipolar disorder,^[^ ^18c^ ^]^ anxiety,^[^ ^18d,e^ ^]^ and schizophrenia.^[^ ^18f,g^ ^]^ The relationship between oligodendrocytes and depression has been drawing increasing attention. Abnormal myelin integrity appears at the early stage of depression, and worsen as the disease progressed.^[^ ^8a^ ^]^ Neurotransmitters,^[^ ^18^, ^26^ ^]^ microRNAs,^[^ ^27^ ^]^ regulatory factors,^[^ ^18^, ^28^ ^]^ and inflammatory cytokines^[^ ^29^ ^]^ from oligodendrocytes have been implicated in the pathology of depression. In our study, according to the evidence obtained from different mouse models, we conclude that the oligodendrocytes dysfunction would be sufficient for the presence of depression. Using the chemogenetic mouse model, we illustrate that inhibition of oligodendrocytes activity leads to depressive‐like behaviors. And with the *Olig1^cre/+^;Ninj2^fl/fl^* mice, we found that increased oligodendrocyte necroptosis could also be a cause for depression. Notably, when myelination in *Olig1^cre/+^*;*Ninj2^fl/fl^* mice was restored by Nec‐1s treatment, the depressive‐like behaviors in these mice synchronically ameliorated. Our results provided additional evidence to demonstrate the importance of oligodendrocytes in the pathology of depression.

Nowadays, the communication between oligodendrocytes and neurons has become a popular topic. Previously, we found that oligodendrocytes could regulate neuronal activities and controlled food intake by its lactate production.^[^ ^30^ ^]^ For depression, as mentioned above, a few small molecules produced from oligodendrocytes have been implicated in the pathology of depression.^[^ ^18^ ^]^ In our study, after *Ninj2* ablation, among the TNF*α* downstream cytokines, *Ccl2* was the most significantly induced gene in oligodendrocytes. Treatment of Ccl2 significantly reduced the neuronal activities. Consistent with our results, Ccl2 has been reported to modulate neural activities in several kinds of neurons.^[^ ^24^ ^]^ Therefore, we believed that Ccl2 mediated the signal delivery, at least partially, from oligodendrocytes to neurons that control the presence of depression. CCR2 was the receptor for Ccl2 in neurons,^[^ ^31^ ^]^ and it is a G protein‐coupled receptor that binds G*α*i.^[^ ^32^ ^]^ Recruitment of Ccl2 to CCR2 inhibited intracellular cAMP levels, which activates its downstream protein kinase A signaling pathway and plays a significant role in the development of depressive disorders.^[^ ^33^ ^]^ Therefore, the Ccl2‐CCR2‐cAMP axis could be a potential machinery for the control of neuronal activities and the presence of depression. Besides Ccl2, we could not exclude the possibility that other molecules might also participate in this conversation, such as other cytokines, neurotransmitters, or even nutrients.

It was worth mentioning that we observed dysmyelination and depressive‐like behaviors simultaneously in the *Ninj2*‐deficient mice. Improper myelination was associated with impaired motor function.^[^ ^34^ ^]^ Although we did not find any difference between the WT and mutant mice in the total walking distance in the open field test (Figure S3, Supporting Information), we still could not completely rule out the possibility that the motor impairment caused by dysmyelination was at least partially associated with the poor performances in TST and FST. Interestingly, although the communication between oligodendrocytes and neurons should exist widely, the *Ninj2*‐deficient mice only exhibited apparent depressive‐like behaviors, but not memory or recognition defects, or anxiety. The cause of the quite unique phenotype in the *Ninj2*‐deficient mice is still an open question. The oligodendrocytes in the depression‐relevant regions, such as PFC and hippocampus, might have specified properties when communicating with their nearby neurons. And the neurons in these regions might also have specified susceptibilities to the molecules secreted by their nearby oligodendrocytes. These remaining questions are worthy for further studies to better show the specificity of oligodendrocytes/neurons communication.

Taken together, in this study, we identified Ninj2 in oligodendrocytes as an inhibitory factor in the development of depression. Our finding revealed a potential therapeutic target for depression, and further emphasized the significance of oligodendrocytes in the development of depression.

## 4 Experimental Section

### Mice

The *Ninj2^fl/fl^* mice were generated by Shanghai Biomodel Organism Science and Technology Development Company. The Gt(ROSA)26Sor^tm1(CAG‐CHRM4*,‐mCitrine)Ute^/J mice were purchased from the Jackson laboratory (Cat# 026219). C57BL/6 mice and Sprague–Dawley rats were purchased from Xiamen University Laboratory Animal Center. All mice were maintained in the Xiamen University Laboratory Animal Center. All of the animal experiments were approved by and performed according to the experimental guidelines of the Animal Care and Use Committee of Xiamen University.

### Antibodies

The antibodies used in immunofluorescence and western blot included Ninj2 (Cat# AF5056, R&D systems), MBP (Cat# SMI‐94R, Covance), Olig2 (Cat# AB9610, Millipore), CC1 (Cat# OP80, Calbiochem), PDGFR*α* (Cat# SC‐338, Santa Cruz Biotechnology), Cleaved‐caspase 3 (Cat# 9661, Cell Signaling Technology), phosphorylated MLKL (Cat# ab196436, abcam), MLKL (Cat# 66675‐1‐lg, proteintech), TNF*α* (Cat# 3707, Cell Signaling Technology), BrdU (Cat# ab6326, abcam), Ki67 (Cat# GTX16667, Gene Tex), CNP (Cat# 5664, Cell Signaling Technology), GAPDH (Cat# 60004‐1‐lg, Proteintech), HA (Cat# SC‐7392, Santa Cruz Biotechnology), Flag (Cat# F‐1804, Sigma), and 488/555 donkey anti‐mouse/rabbit/rat secondary antibodies (Invitrogen).

### Mouse Oligodendrocyte Precursor Cell Primary Culture

Mouse OPCs were isolated from the cortices of pups at postnatal days 3–8 as described previously.^[^ ^30^ ^]^ Briefly, cortical tissues were dispersed into single cells and the cell suspension was then subjected to immunopanning with antibodies against GalC and O4 sequentially. The enriched Galc‐negative O4‐positive OPCs were plated into poly‐D‐lysine coated dishes and cultured with Mouse OPC growth medium (DMEM/F‐12 (GIBCO, Cat# 11330‐032) supplemented with 1% N2 supplement (GIBCO, Cat# A1370701), 2% B27 supplement (GIBCO Cat# A3582801), penicillin–streptomycin solution (MP Biomedicals, Cat# 0916700), 1% sodium pyruvate (GIBCO, Cat# 11360070), 1% L‐glutamine (Hyclone, Cat# SH30034), 10 ng mL^−1^ platelet‐derived growth factor‐aa (Peprotech, Cat#100‐13A), 10 ng mL^−1^ ciliary neurotrophic factor (Peprotech, Cat# 450‐13), 20 ng mL^−1^ human basic fibroblast growth factor (Sino Biological, Cat# 10014HNAE), 0.5 mg mL^−1^ insulin (Sigma, Cat# 91077), 5 mg mL^−1^ *N*‐acetyl cysteine (Sigma, Cat# A8199), 10 ng mL^−1^ D‐biotin (Sigma, Cat# B4639), 5 mm forskolin (Sigma, Cat# F3917), and 0.1% Trace Elements B (Corning, Cat# 25‐022‐CI)). OPC differentiation medium contained the same components as growth medium except human basic fibroblast growth factor and platelet‐derived growth factor‐aa, but supplemented with 40 ng mL^−1^ triiodo‐thyronine (Sigma, Cat# T2877).

### Rat Oligodendrocyte Precursor Cell Primary Culture

Rat OPCs were isolated from the cortices of pups at postnatal days 0–2 using a differential detachment procedure as described previously.^[^ ^35^ ^]^ The isolated OPCs were grown in rat OPC growth medium (DMEM/F‐12 medium supplemented with 1% penicillin–streptomycin, 1% N2, 2% B27, 10 ng mL^−1^ platelet‐derived growth factor‐aa and 20 ng mL^−1^ human basic fibroblast growth factor) and differentiated in oligodendrocyte differentiation medium (DMEM/F‐12 medium supplemented with 1% penicillin–streptomycin, 1% N2, 2% B27, 10 ng mL^−1^ ciliary neurotrophic factor, 40 ng mL^−1^ triiodo‐thyronine) for 3 days to maturation.

### Mouse Cortical Neuron Primary Culture

Primary neurons were dissected from E16.5 mouse embryos as described previously.^[^ ^36^ ^]^ Briefly, brain cortices were dissected, the meninges were removed, and cortical tissue was dissociated by enzymatic digestion. Isolated primary cortical neurons were plated on poly‐D‐lysine coated dishes, and cultured in Neurobasal medium supplemented with 1% penicillin–streptomycin, 1% L‐glutamine, 2% B27, and maintained in a 5% CO_2_ incubator at 37 °C.

### Immunofluorescence

Mouse brains were perfused with 4% paraformaldehyde (PFA) followed by dehydration with 30% sucrose, then 16 µm frozen sections were prepared. Mouse brain sections or cultured cells were incubated in blocking solution for 1 h at room temperature. Primary antibodies were applied overnight at 4 °C. The next day, sections were incubated with the secondary antibodies for 2 h at room temperature. Images were acquired using a confocal microscope (Leica SP8). The fluorescence intensity analysis and cell counting were performed using ImageJ software.

### TUNEL

DeadEnd Fluorometric TUNEL System (Promega, Cat# G3250) was used by following manufacture's protocol to detect cell death.

### Western Blotting Analysis

The tissues were isolated and then lysed in RIPA buffer (50 mm Tris‐HCl, pH 7.5, 150 mm NaCl, 1 mm EDTA, 1% Triton X‐100, 1% deoxycholate, 0.1% SDS), and the cells were lysed in cell lysis buffer (20 mm Tris‐HCl, pH 7.5, 150 mm NaCl, 1 mm EDTA, 1 mm EGTA, 1% Triton X‐100, 2.5 mm sodium pyrophosphate, 1 mm b‐glycerophosphate, 1 mm NaF, 1 mm PMSF), supplemented with protease inhibitor cocktail (MCE, Cat# HY‐K0010), phosphatase inhibitor cocktail I (MCE, Cat# HY‐K0021), and phosphatase inhibitor cocktail II (MCE, Cat# HY‐K0022) on ice for 30 min, followed by centrifugation at 12 000 rpm for 30 min. Protein concentrations were measured using the BCA Protein Assay Kit (Sangon Biotech, Cat# C503021). Proteins were loaded on SDS‐PAGE gel and transferred to PVDF membrane (GE Healthcare, Cat# 10600023). Membranes were blocked with 3% BSA in TBS (50 mm Tris‐HCl, pH 7.5, 150 mm NaCl) for 1 h and then incubated overnight at 4 °C with primary antibodies. After three washes with TBS plus 0.05% Tween 20, membranes were incubated with secondary antibodies for 1 h and detected using an ECL kit (Millipore, Cat# WBKLS0500). ImageJ software was used to quantify the signal intensity in western blot assay.

### Real‐Time Quantitative PCR

Total RNA was extracted using TRIzol (Takara, Cat # 9109) according to the manufacturer's instructions. cDNAs were prepared from 1 µg total RNA using Hifair II 1st Strand cDNA Synthesis SuperMix for qPCR (Yeasen, Cat# 11123ES60) according to the manufacturer's instructions. Real‐time quantitative PCR was performed using Hieff qPCR SYBR Green Master Mix (Yeasen, Cat# 11201ES08). Primers used are listed in Table S1, Supporting Information.

### RNA‐Seq Analysis

RNA‐Seq analysis with mouse oligodendrocytes from wild type or *Olig1^cre/+^;Ninj2^fl/fl^* mice cortices (two samples/genotype) were performed by Novogene (Beijing, China). All RNA‐Seq data were aligned to Mm10 by Hisat2 v2.0.5. FeatureCounts v1.5.0‐p3 and StringTie (v1.3.3b) were used to generate gene counts. Differential expressed genes were identified using DESeq2 with fold change > 2 and *p* < 0.05.

### Co‐Immunoprecipitation

Cells were transfected with plasmid for 48 h, then lysed and incubated with anti‐HA antibodies overnight at 4 °C. Then the complexes were precipitated with 20 µL of Protein A Magnetic Beads (MCE, Cat# HY‐K0203) with gentle agitation at 4 °C for 3 h. The beads were washed, and the immunoprecipitated protein complex was processed for western blotting.

### Nucleofection of Rat Oligodendrocyte Precursor Cells

Nucleofection of rat OPCs was performed using the Amaxa Nucleofector protocol (Lonza). Briefly, the rat OPCs were harvested by trypsinization. The cell pellet was resuspended carefully in 100 µL Nucleofector Solution per sample. Plasmids (pcDNA3.3‐HA‐TNFR1/plvo3‐Flag‐Ninj2) were added to cell suspension. The mixture was subjected to nucleofection using Nucleofector Program O‐017 in a certified cuvette. After nucleofection, 500 µL of the pre‐equilibrated rat OPC growth medium were immediately added into the cuvette, and the samples were gently transferred into the prepared plate. After 48 h, the cells were lysed and incubated with anti‐HA antibodies, followed with co‐immunoprecipitation methods.

### Enzyme Linked Immunosorbent Assay

Ccl2 levels in cell culture supernatant were measured using mouse Ccl2 enzyme linked immunosorbent assay (ELISA) Kits (Boster, Cat# EK0568) according to the manufacturer's instructions.

### Nec‐1s Administration

Nec‐1s (MCE, Cat# HY‐14622A) was dissolved in DMSO, and then transferred into 0.9% NaCl. Mice at P60 were intraperitoneally injected with Nec‐1s (10 mg kg^−1^) daily for 30 days, as described in published studies.^[^ ^19^, ^25^ ^]^

### Clozapine‐*N*‐Oxide Treatment

The* Gi‐DREADD* or *Olig1^cre/+^;Gi‐DREADD* mice at P60 were intraperitoneally injected with CNO (2 mg kg^−1^), and the animal behaviors were tested 2 h after injection as described previously.^[^ ^30^, ^37^ ^]^

### Electron Microscopy

Mice were perfused with fixed buffer (4% PFA, 0.5% glutaraldehyde, 0.01 m phosphate buffer, pH 7.4), the tissues were dissected and fixed immediately in a fixative solution (2.5% glutaraldehyde, 0.01 m phosphate buffer, pH 7.4) at room temperature for 2 h, and then at 4 °C overnight. The ultrathin sections were investigated under a transmission electron microscope (Hitachi HT‐7800). Myelinated axon numbers were analyzed using ImageJ software, and G‐ratio were analyzed using Image Pro Plus software.

### Golgi Staining

Golgi staining was performed using the FD Rapid Golgi Stain kit according to the manufacturer's instructions (FD Neuro Technologies, INC). Briefly, mice brains were immersed in the impregnation solution, comprising equal volumes of Solutions A and B, and stored at room temperature for 2 weeks in the dark. Then the brains were transferred into Solution C and stored at 4 °C in the dark for at least 48 h. The brains were sectioned using a vibratome (Leica 1200S) at a thickness of 150 µm. The sections were then stained with Solutions D and E. Golgi‐stained hippocampal CA1 pyramidal neurons and the dendritic segments were imaged by microscopy (Olympus FV1000).

### Analysis of Dendritic Complexity and Spine Density

As described previously,^[^ ^38a,b^ ^]^ dendritic complexity and apical distal spine numbers were analyzed using ImageJ software. Dendritic intersection analysis was measured using the Sholl analysis plugin in ImageJ. For Sholl analysis, the dendritic trees were traced and reconstructed using the NeuronJ plugin in ImageJ, and then the traced dendritic trees were converted into a binary image. Sholl analysis parameters were as follows: starting radius, 10 µm; ending radius, 200 µm; radius step size, 10 µm. For spine density quantification, the apical distal dendrite was traced and its length was measured by ImageJ software, and then the number of spines was counted.

### Whole‐Cell Patch‐Clamp Recordings

Mice were anesthetized with isoflurane, and brains were rapidly removed and placed in ice‐cold, high‐sucrose cutting solution (120 mm sucrose, 64 mm NaCl, 26 mm NaHCO_3_, 10 mm glucose, 2.5 mm KCl, 1.25 mm NaH_2_PO_4_, 10 mm MgSO_4_, and 0.5 mm CaCl_2_, aerated with 95% O_2_ and 5% CO_2_). 400 µm thick transverse slices were sectioned on a vibratome (Leica 1200S) in high‐sucrose cutting solution and immediately transferred to an incubation chamber with artificial cerebrospinal fluid (ACSF) (126 mm NaCl, 2.5 mm KCl, 1.2 mm NaH_2_PO_4_, 2.4 mm MgCl_2_·6H_2_O, 1.2 mm CaCl_2_, 18 mm NaHCO_3_, and 11 mm glucose, aerated with 95% O_2_ and 5% CO_2_). Slices were allowed to recover at 32 °C for 30 min and then equilibrated at room temperate for 30 min. During recordings, brain slices or primary cultured neurons were placed in a recording chamber perfused with ACSF and continuously aerated with 95% O_2_ and 5% CO_2_. All recordings were performed in the prefrontal cortex or hippocampal CA1 pyramidal neurons, or primary cultured pyramidal neurons, which were identified by size and morphology. Recording electrodes were made from borosilicate glass with a resistance 5–8 MΩ when filled with intracellular solution (140 mm CsCH_3_SO_3_, 2 mm MgCl_2_·6H_2_O, 5 mm TEA‐Cl, 10 mm HEPES, 1 mm EGTA, 2.5 mm Mg‐ATP, and 0.3 mm Na_2_‐GTP [pH 7.2–7.4]). Recordings were obtained using a MultiClamp 700B amplifier (Molecular Devices), filtered at 2 kHz, and digitized at 10 kHz using Digidata 1550B (Molecular Devices). In the presence of ACSF supplemented with 1 µm tetrodotoxin, mEPSCs were recorded at a holding potential of −70 mV, and mIPSCs were recorded at 0 mV. The recorded mESPCs and mIPSCs were analyzed using ClampFit (Molecular Devices) and MiniAnalysis (Synaptosoft) software.

### Behavior Tests

For all behavioral tests, adult male mice were used.

### Rotarod Test

Motor function and balance ability were evaluated by rotarod apparatus. Mice were placed on a rotarod (Ugo Basile). The rotarod was constantly accelerated from 5 to 40 rpm and the latency to fall was recorded. The procedure was repeated for five trials, which was averaged to give the latency to fall for each mouse.

### Forelimb Grip Strength

The grip strength meter (Ugo Basile) was used to measure the forelimb grip strength. The mice were held by the base of the tail and allowed to freely grasp a grid that was connected to a force transducer with only their front paws, and were steadily pulled back horizontally. Each mouse was measured for five trials, and the average force was recorded.

### Forced Swimming Test

Mice were placed in a clear cylinder filled with water (23 ± 1 °C) for 6 min and recorded by a video tracking system (Smart 3.0). Immobility time in the total 6 min was measured.

### Tail Suspension Test

Mice were suspended by their tails from a tail suspension frame for 6 min and monitored using a video tracking system (Smart 3.0). Escape‐related behavior was assessed, and immobility time during the 6 min suspension period was recorded.

### Sucrose Preference Test

Mice were trained to consume 1% sucrose and water from two different bottles. Sucrose and water bottles were placed on randomly assigned sides of the cage, and were switched to eliminate side preference. Then mice were allowed free access to 1% sucrose and water from two differing bottles, the total weight of liquid consumed from each bottle was measured after 24 h. Sucrose preference was calculated according to the following formula: Sucrose preferences (%) = sucrose consumption/(sucrose + water consumption) × 100%.

### Y Maze Test

Y maze test was carried out in a three arms horizontal maze. Mice moved freely through the maze for 5 min, recorded by a video tracking system (Smart 3.0). Spontaneous alternation was defined as successive entries into the three arms.

### Elevated Plus Maze Test

The elevated plus maze consisted of two open and two closed arms. Mice activities were tracked for 5 min with a video tracking system (Smart 3.0). The time spent in the open arms was measured.

### Open Field Test

Mice were placed in the center of an open field box, and their activities were recorded for 10 min with a video tracking system (Smart 3.0). The time spent in the center zone, as well as the total distances traveled, were measured.

### Novel Object Recognition Test

The test was carried out in an open box containing two identical objects. Mice were placed in the center of the apparatus and explored for 5 min. 2 h later, one of the objects was replaced by a novel object with different form and color, and the exploratory activity was recorded for 5 min by a video tracking system (Smart 3.0). The novel object recognition index was determined as the ratio of the time spent in exploring the novel object to the total time spent in exploring both objects.

### Water Maze

The water maze was divided into four quadrants, mice were trained to escape from water by swimming to the platform, and recorded by a video tracking system (Smart 3.0). All mice were trained four times daily for 4 consecutive days. On day 5, the platform was removed and a probe test was conducted. The time spent in the platform‐located quadrant, distance traveled in the platform quadrant, or times crossing the platform area was measured.

### Statistical Analysis

The sample sizes (*n*) were indicated in the figure legends. For animal behavior tests, the data were automatically captured and analyzed by an automatic analysis program. All statistical analyses were performed using GraphPad Prism version 8.0. Data were present as Mean ± SEM. A two‐tail test was chosen for the cases using an unpaired *t*‐test. For cases using one‐way ANOVA or two‐way ANOVA, the Tukey's multiple comparisons tests were performed for posthoc tests. *p* values were indicated with single asterisk (*p < 0.05), double asterisks (**p < 0.01), triple asterisks (***p < 0.001), and quadruple asterisk (****p < 0.0001) on graphs. Data collected were normalized to the control group. No data were excluded from statistical analysis.

## Conflict of Interest

The authors declare no conflict of interest.

## Author Contribution Statement

Y.S., X.C., and Z.O. contributed equally to this work, conceived and performed experiments, and wrote the manuscript. C.L. and Y.C. conceived experiments and wrote the manuscript. T.Z. wrote the manuscript. Y.W. and W.C. performed experiments.

## Supporting information

Supporting Information

Supplemental Table 1

## Data Availability

The data that support the findings of this study are available from the corresponding author upon reasonable request.
