# Supplementary material for: Dysmyelination by Oligodendrocyte‐Specific Ablation of Ninj2 Contributes to Depressive‐Like Behaviors
Source: Adv Sci (Weinh). 2021 Nov 17;9(3):2103065. doi: 10.1002/advs.202103065 (PMC8787401; doi:10.1002/advs.202103065)
Supplement: Supplementary file 1 — Supporting Information [file ADVS-9-2103065-s001.pdf]

ADVANCED SCIENCE
Open Access

Supporting Information

for *Adv. Sci.*, DOI: 10.1002/advs.202103065

Dysmyelination by oligodendrocyte-specific ablation of *Ninj2* contributes to depressive-like behaviors

Yuxia Sun, Xiang Chen, Zhimin Ou, Yue Wang, Wenjing Chen, Tongjin Zhao, Changqin Liu, Ying Chen**

Supporting Information

Dysmyelination by oligodendrocyte-specific ablation of *Ninj2* contributes to depressive-like behaviors

Yuxia Sun, Xiang Chen, Zhimin Ou, Yue Wang, Wenjing Chen, Tongjin Zhao,

Changqin Liu[*], *Ying Chen*[*]

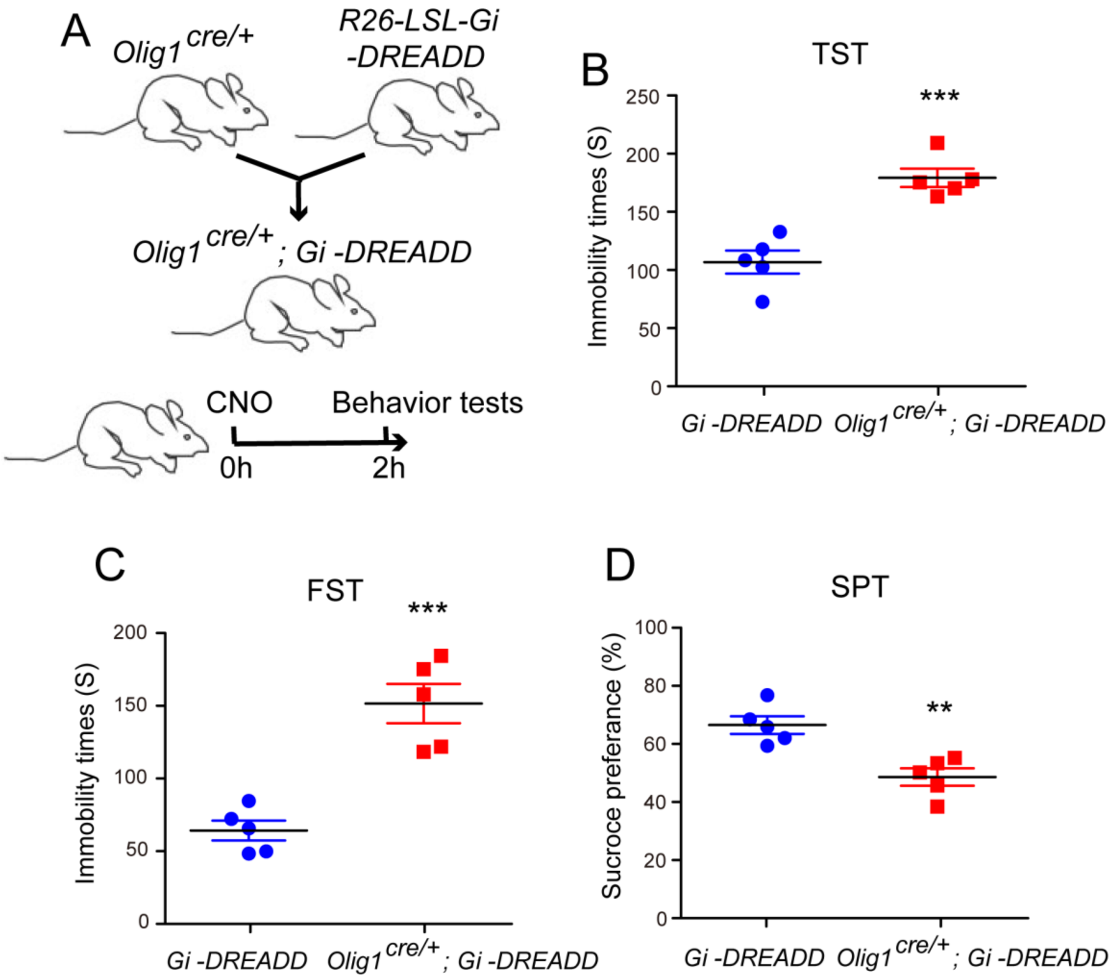

Supplementary Figure 1. Oligodendrocytes play a role in depression. Related to Figure 1.

(A) The strategy of generating *Olig1*$^{cre/+}$*;Gi-DREADD* mice, and the timeline of the behavioral tests. (B-D) Tail suspension test (TST), force swimming test (FST) and sucrose preference test (SPT) were performed in CNO-treated *Gi-DREADD* and *Olig1*$^{cre/+}$*;Gi-DREADD* mice, $n = 5$ mice/genotype. All the quantification data are presented as mean ± SEM, *p*-values are calculated using two-tailed unpaired Student's t-test, ** $p < 0.01$, *** $p < 0.001$.

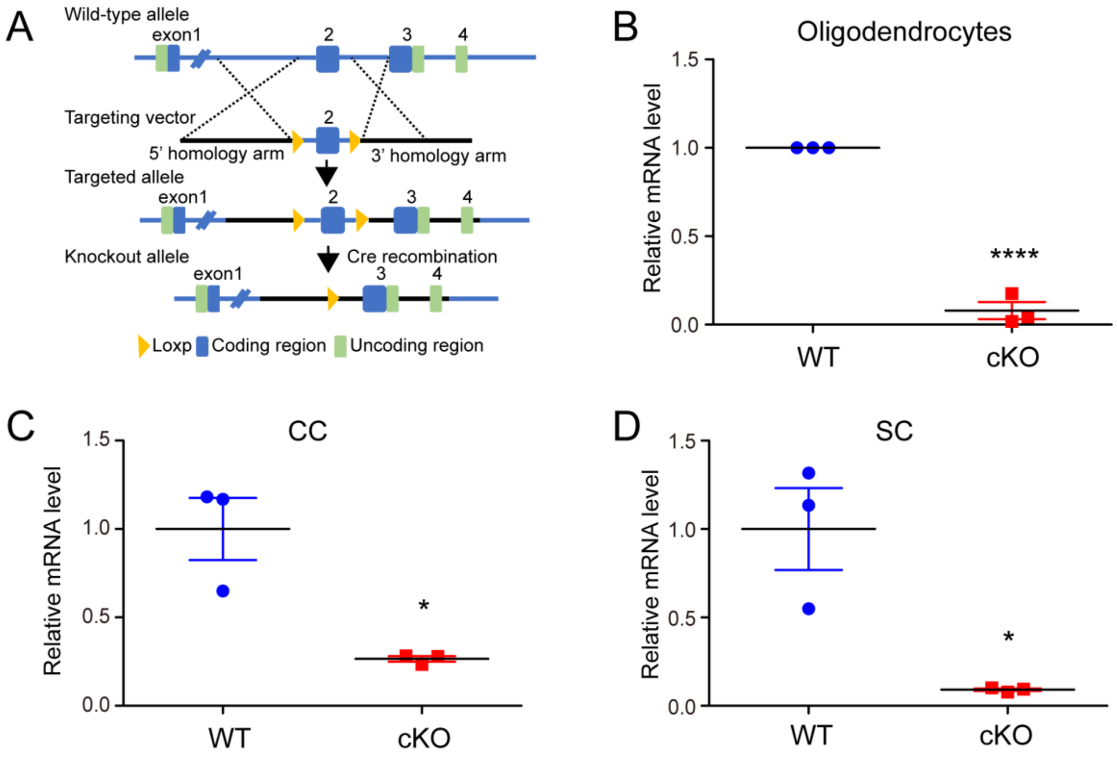

Supplementary Figure 2. Confirmation of *Ninj2* knockout in oligodendrocytes, corpus callosum and spinal cord. Related to Figure 1.

(A) The strategy of generating *Olig1*$^{cre/+}$;*Ninj2*$^{fl/fl}$ and *Cnp*$^{cre/+}$;*Ninj2*$^{fl/fl}$ mice. (B-D) Real-time PCR analysis on the mRNA level of *Ninj2* in oligodendrocytes (B) (n = 3 independent experiments), corpus callosum (CC) (C) and spinal cord (SC) (D) from WT or *Olig1*$^{cre/+}$;*Ninj2*$^{fl/fl}$ (cKO) mice (n = 3 mice/genotype). All the quantification data are presented as mean ± SEM, p-values are calculated using two-tailed unpaired Student's t-test, * $p < 0.05$, **** $p < 0.0001$.

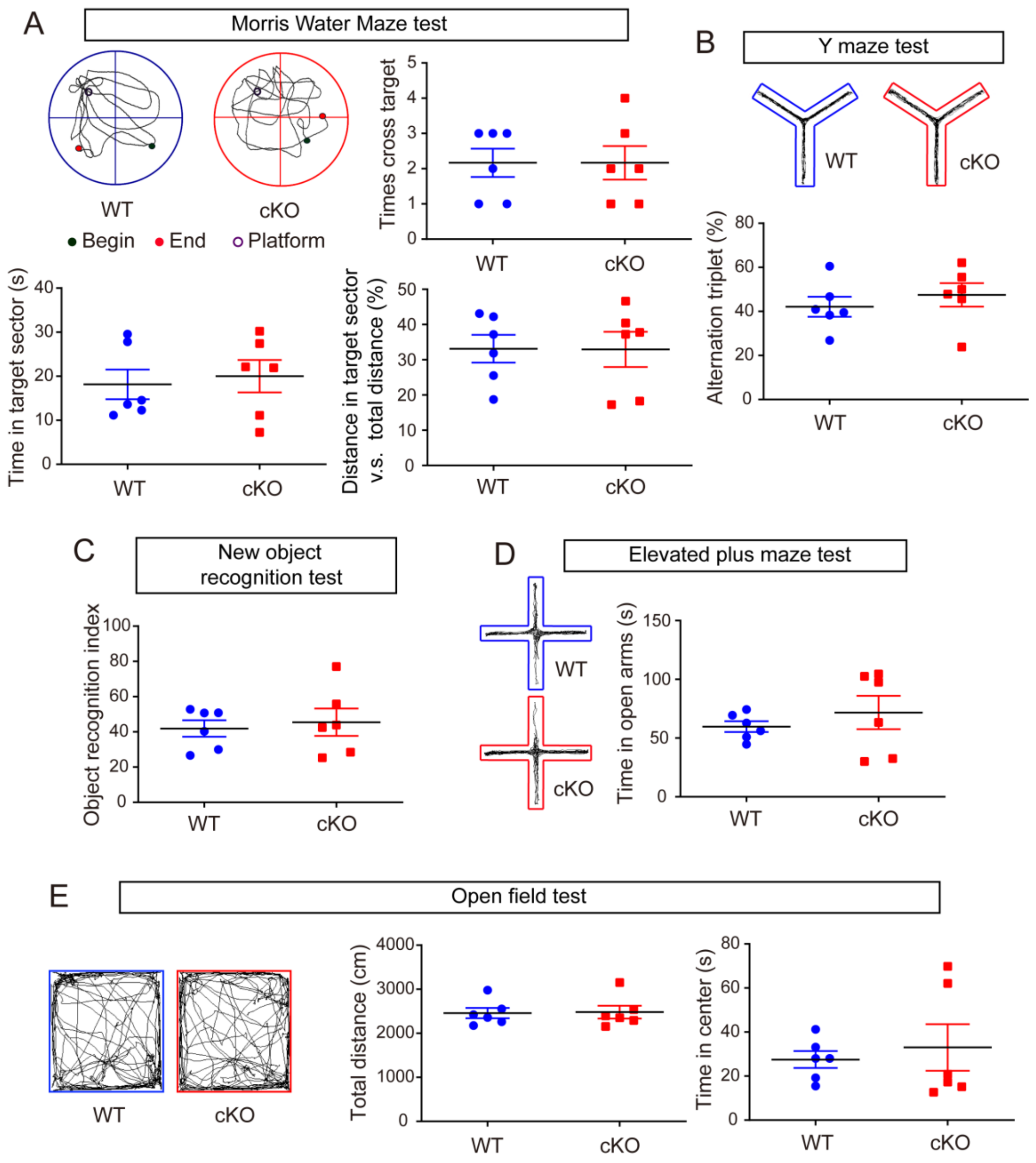

Supplementary Figure 3. Loss of *Ninj2* in oligodendrocytes has no effect on memory, recognition, or anxiety-like behaviors. Related to Figure 1.

WT and *Olig1*$^{cre/+}$;*Ninj2*$^{fl/fl}$ (cKO) mice at P60 were subjected to (A) Morris water maze test, (B) Y maze test, (C) Novel object recognition test, (D) Elevated plus maze test, (E) Open field test to evaluate their status on memory, recognition, or anxiety-like behaviors. *n* = 6 mice/genotype. All the quantification data are presented

as mean ± SEM, *p*-values are calculated using two-tailed unpaired Student’s t-test.

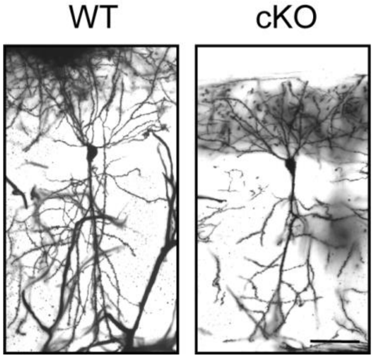

Supplementary Figure 4. Loss of *Ninj2* reduces dendritic complexity of pyramidal neurons in the hippocampus CA1 area. Related to Figure 1E.

Golgi staining of WT and *Olig1*$^{cre/+}$;*Ninj2*$^{fl/fl}$ (cKO) mice at P60. Scale bar, 50 μm.

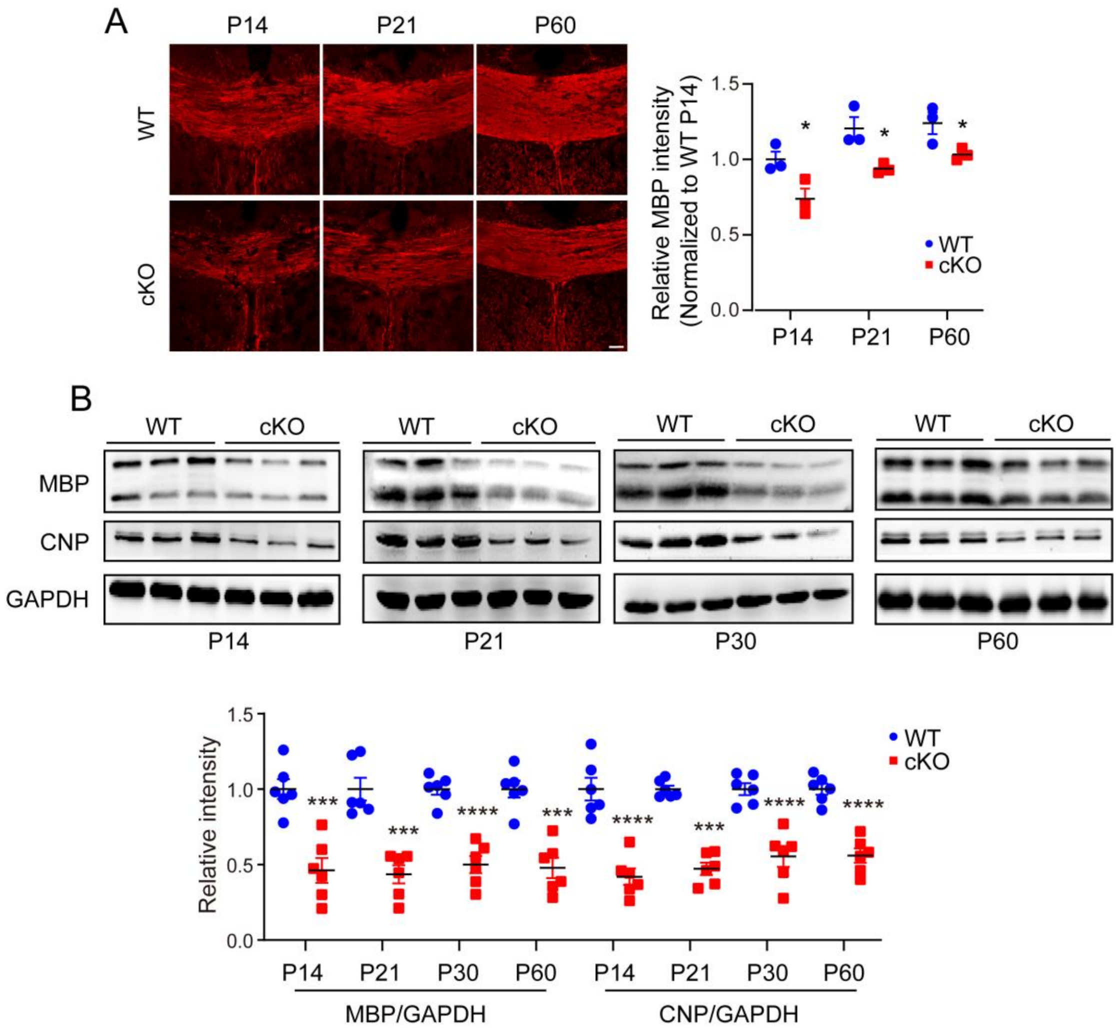

Supplementary Figure 5. Loss of *Ninj2* reduces the expression of myelin-related proteins in corpus callosum. Related to Figure 1.

(A) Immunofluorescent visualization and quantification of MBP expression in the corpus callosum from WT or *Olig1*$^{cre/+}$;*Ninj2*$^{fl/fl}$ (cKO) mice at P14, P21 and P60, respectively. *n* = 3 mice/genotype. (B) Western blot analyses of MBP and CNP in the corpus callosum from WT or cKO mice at P14, P21, P30 and P60, respectively. *n* = 6 mice/genotype. All the quantification data are presented as mean ± SEM, *p*-values are calculated using two-tailed unpaired Student's t-test, * $p < 0.05$, *** $p < 0.001$, **** $p < 0.0001$.

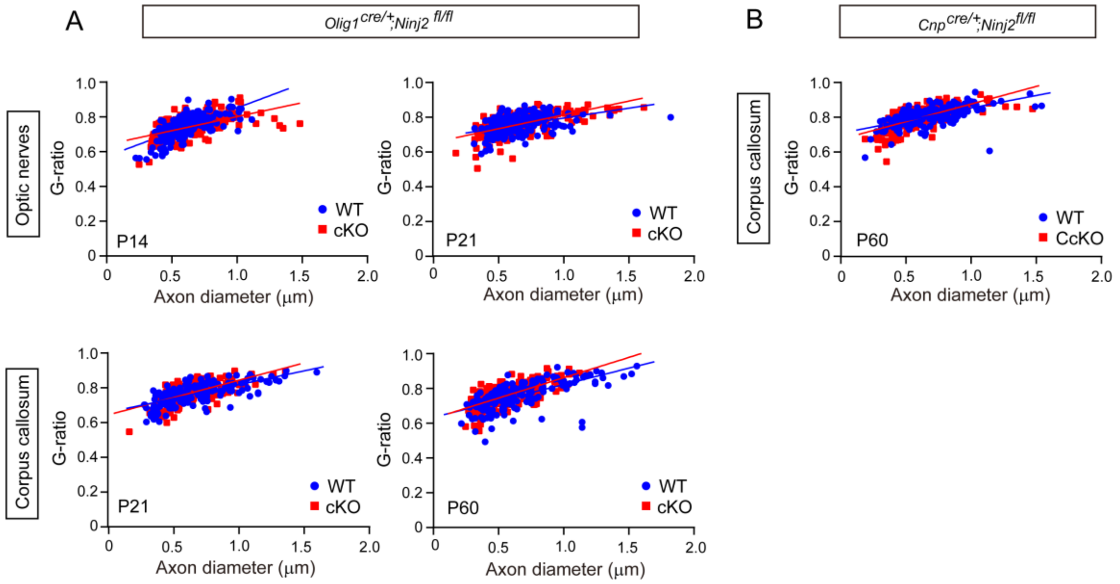

Supplementary Figure 6. Loss of *Ninj2* has no effect on myelin thickness. Related to Figure 2.

(A-B) G-ratio of the optic nerve at P14 and P21, the corpus callosum at P21 and P60 from WT or *Olig1*$^{cre/+}$;*Ninj2*$^{fl/fl}$ (cKO) mice. (C) G-ratio of the corpus callosum at P60 from WT or *Cnp*$^{cre/+}$;*Ninj2*$^{fl/fl}$ (CcKO) mice. *n* = 3 mice/genotype, at least 50 axons/mouse had been analyzed. All the quantification data are presented as mean ± SEM, *p*-values are calculated using two-tailed unpaired Student's t-test.

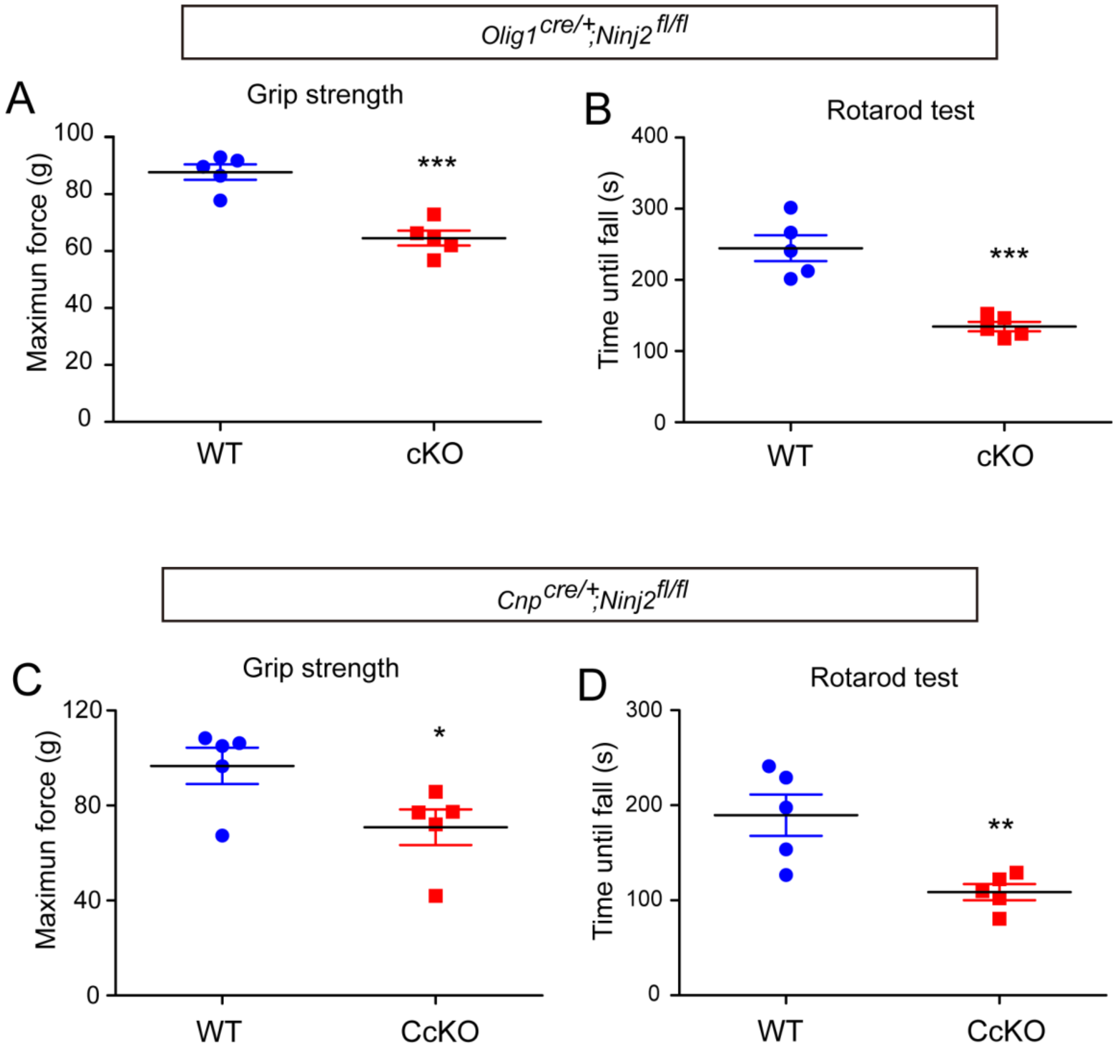

Supplementary Figure 7. Loss of *Ninj2* leads to motor defect in mice. Related to Figure 2.

(A-B) Forelimb grip strength and rotarod test on WT or *Olig1*$^{cre/+}$;*Ninj2*$^{fl/fl}$ (cKO) mice at P60. (C-D) Forelimb grip strength and rotarod test on WT or *Cnp*$^{cre/+}$;*Ninj2*$^{fl/fl}$ (CcKO) mice at P60. $n = 5$ mice/genotype. All the quantification data are presented as mean ± SEM, *p*-values are calculated using two-tailed unpaired Student’s t-test. * $p < 0.05$, ** $p < 0.01$, *** $p < 0.001$.

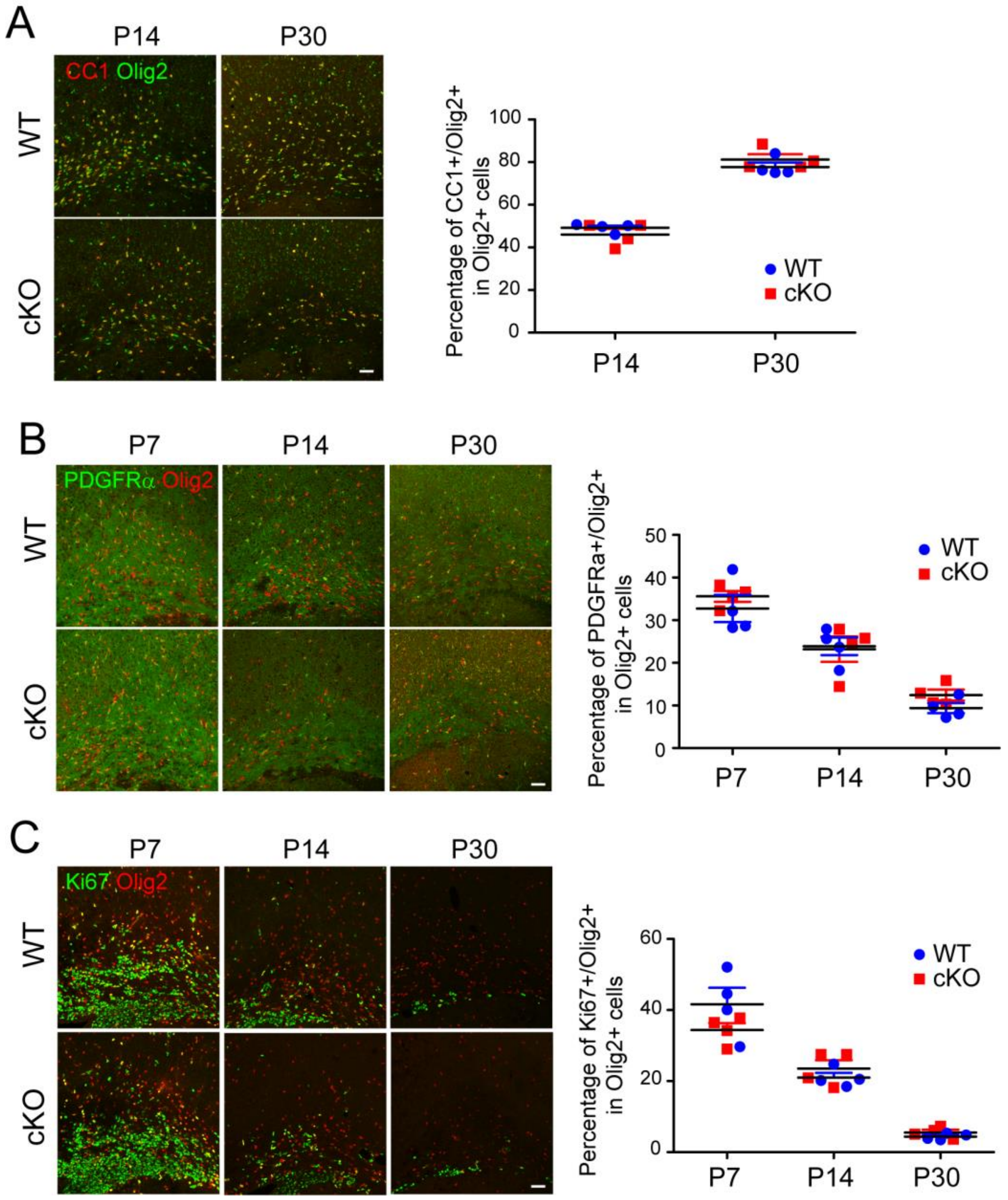

Supplementary Figure 8. Loss of *Ninj2* has no effect on oligodendrocyte proliferation or differentiation. Related to Figure 3.

(A-C) Immunofluorescent staining against CC1 (A), PDGFRα (B), and Ki67 (C), were performed in the corpus callosum sections from WT or $Olig1^{cre/+}$;$Ninj2^{fl/fl}$ (cKO) mice at P7, P14 and P30, the percentages of the double-positive cells in total $Olig2^{+}$ cells were quantified and shown on the right panels. Scale bar, 50 μm. n = 4

mice/genotype, All the quantification data are presented as mean ± SEM, *p*-values are calculated using two-tailed unpaired Student's t-test.

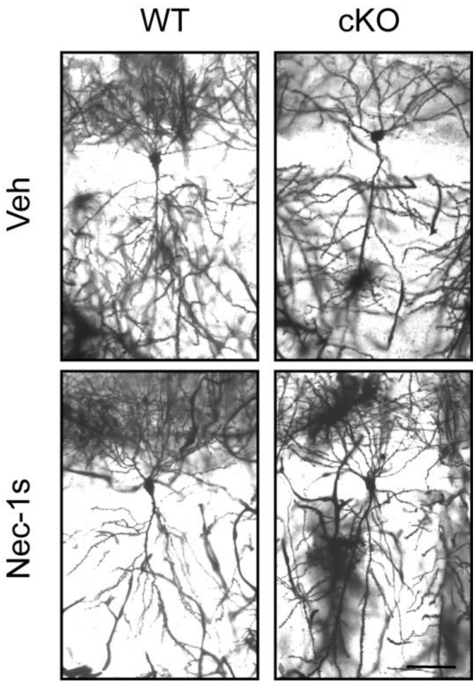

Supplementary Figure 9. Nec-1s treatment restores dendritic complexity of pyramidal neurons in the hippocampus CA1 area of *Ninj2*-deficient mice. Related to Figure 7A.

Golgi staining of WT or $Olig1^{cre/+}$;$Ninj2^{fl/fl}$ (cKO) mice at P60, which received i.p. injection with vehicle or Nec-1s (10 mg/kg) from P60 to P90. Scale bar, 50 µm.
